# Assessing the survival advantage of deferred cytoreductive nephrectomy using a prediction interval

**DOI:** 10.1097/JS9.0000000000001008

**Published:** 2023-12-21

**Authors:** Kuo-Chuan Hung, I-Ting Tsai, Cheuk-Kwan Sun

**Affiliations:** a Department of Anesthesiology, Chi Mei Medical Center, Tainan City; b Department of Emergency Medicine, E-Da Hospital; c School of Medicine, College of Medicine; d Department of Emergency Medicine, E-Da Dachang Hospital, I-Shou University, Kaohsiung City, Taiwan

*Dear Editor,*

We read with great interest the recent article by Li *et al*.^1^ comparing upfront versus deferred cytoreductive nephrectomy in patients with metastatic renal cell carcinoma receiving systemic therapy. The authors conducted a systematic review and meta-analysis of nine studies including over 3000 patients^1^. They found that deferred cytoreductive nephrectomy (dCN) was associated with superior overall survival compared to upfront cytoreductive nephrectomy (uCN). Despite the importance of such a finding that could influence clinical decision-making for this patient population, there are some limitations that should be considered when interpreting the result (i.e. overall survival). In particular, there was significant heterogeneity in the overall survival across the studies included in the meta-analysis (*I* ^2^=59%)^1^. Although the authors conducted sensitivity analyses, they were unable to identify the source of heterogeneity that may introduce uncertainty into the pooled effect estimates.

To further explore the robustness of the findings, we calculated the 95% prediction interval based on the raw data from the meta-analysis^1^ to capture the expected range of effects around the summary estimate^2,3^. By taking into account both the variation between studies and the uncertainty around the summary effect, a prediction interval indicates the interval within which we would expect the true effect size to lie for about 95% of similar studies that might be conducted in the future. If the prediction interval includes clinically important benefits and harm, it suggests uncertainty about the net benefit or net harm from future studies. Wide prediction intervals indicate substantial variations in treatment effects across different settings. The prediction interval, which was calculated using Comprehensive Meta-Analysis (Version 4, Biostat, Englewood, New Jersey), showed a prediction interval for overall survival of 0.377–1.35 (Fig. 1). Since this interval includes the null value of 1, it suggests considerable uncertainty regarding the pooled effect, even if the overall results were statistically significant in that meta-analysis^1^. In summary, while that study^1^ provided evidence in support of an association between dCN and an improvement in overall survival, its findings should be interpreted with caution given the high heterogeneity and wide prediction interval.

**Figure 1:**
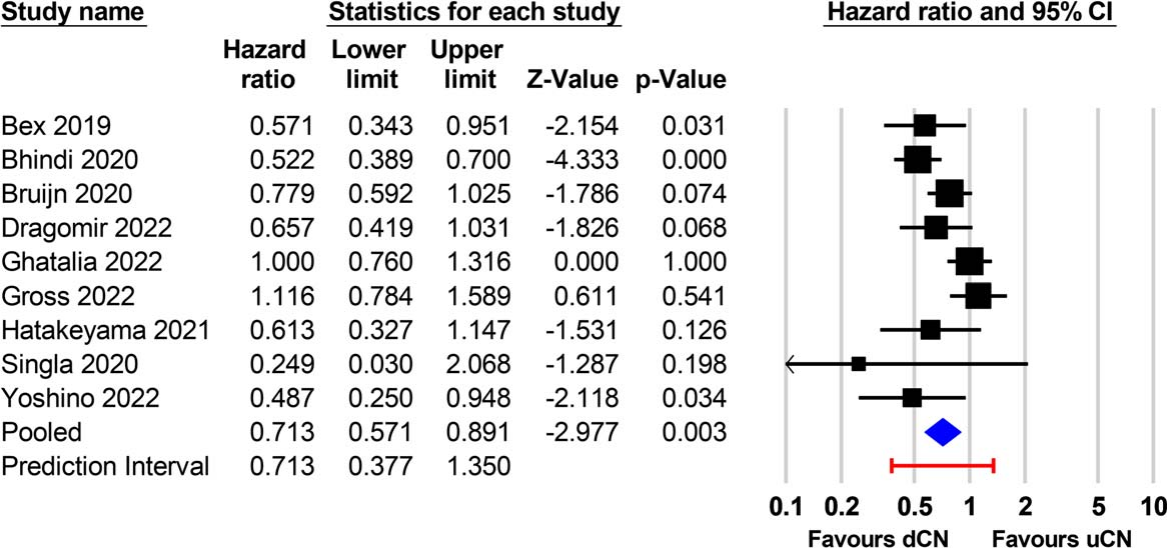
Forest plot of overall survival from meta-analysis with 95% prediction interval. The forest plot shows the hazard ratios and 95% confidence intervals for overall survival from the meta-analysis comparing deferred versus upfront cytoreductive nephrectomy. The diamond represents the pooled hazard ratio of 0.71 with the lateral tips of the diamond indicating the 95% confidence interval (0.57–0.89). The red line below the diamond depicts the 95% prediction interval, ranging from 0.377 to 1.35. Such a wide interval reflects the expected variation in true effects among similar studies. CI, confidence interval; dCN, deferred cytoreductive nephrectomy; uCN, upfront cytoreductive nephrectomy.

## Ethical approval

Not applicable.

## Consent

Not applicable.

## Sources of funding

Not applicable.

## Author contribution

K.-C.H. and C.-K.S.: conceptualization, methodology, and software; K.-C.H. and I-T.T.: data curation; K.-C.H. and C.-K.S.: writing – original draft preparation; K.-C.H. and I-T.T.: visualization and investigation; C.-K.S.: supervision; K.-C.H. and I-T.T.: software and validation; K.-C.H. and C.-K.S.: writing – reviewing and editing.

## Conflicts of interest disclosure

There are no conflicts of interest.

## Research registration unique identifying number (UIN)

Not applicable.

## Guarantor

Kuo-Chuan Hung, I-Ting Tsai, and Cheuk-Kwan Sun.

## Data availability statement

The datasets used and/or analyzed in the current study are available from the corresponding author upon reasonable request.

## Provenance and peer review

This paper was not invited.
